# In Vitro and In Vivo Survey of Ethyl Acetate Extract of *Acorus calamus* (Sweet Flag) Rhizome on *Toxoplasma gondii*

**DOI:** 10.1155/2021/6656023

**Published:** 2021-05-13

**Authors:** Maryam Nikkhoo, Qasem Asgari, Mahmood Reza Moein, Kambiz Yaghoobi, Abbas Gholipour

**Affiliations:** ^1^Department of Pharmacognosy, School of Pharmacy, Shiraz University of Medical Sciences, Shiraz, Iran; ^2^Department of Parasitology and Mycology, School of Medicine, Shiraz University of Medical Sciences, Shiraz, Iran; ^3^Department of Biology, Faculty of Science, Payame Noor University, Tehran, Iran

## Abstract

**Background:**

Toxoplasmosis is a zoonosis disease that can cause a variety range of manifestations in human specially fetus duration and immunodeficiency conditions. Due to toxicity and side effects of current treatment, we evaluated in vivo and in vitro effects of ethyl acetate extract of *Acorus calamus* rhizomes (rootstocks) on *Toxoplasma gondii*.

**Methods:**

The plant, *Acorus calamus,* was collected from Sari, North of Iran in spring season. Ethyl acetate extract was provided from plant rhizomes using Soxhlet apparatus. The total phenolic and flavonoid contents of the extract were measured by the Folin–Ciocalteu method. The mortality effect of different concentrations (1-256 *μ*g/ml) of the extract on *Toxoplasma* tachyzoites was assessed by flowcytometry and propidium iodide staining. For the therapeutic effect assessment, the tachyzoites were inoculated intraperitoneally to mice, and then these mice were orally and intraperitoneally administered different concentrations (32, 64, 128, and 256 mg/kg) of the extract. Also, an infected group received PBS including DMSO 1% as negative control, and an infected group administered sulfadiazine as positive control. For toxicity evaluation of this extract, a group only received dose 256 mg/kg.

**Results:**

The plant extract was rich of phenolic compounds (41.27 ± 0.21 mg/g), whereas it contained fewer amounts of flavonoids (4.79 ± 0.01 mg/g). Results of in vitro experiments showed that there is an inverse relationship between the concentrations and the mortality of the parasites (IC_50_ = 200.01 ± 7.74 *μ*g/ml). The highest percentage (62%) of dead tachyzoites was seen at maximum concentration of the extract. A significant longevity (8.9 days) was belonged to mice orally administered extract dose (256 mg*/*kg/day).

**Conclusion:**

The ethyl acetate extract of *A. calamus* rhizomes had significant anti-*Toxoplasma* activities either in vitro or in vivo. It may be connected to high amount of phenolic compounds. We suggest that the effects of different fractions and the admin types of the extract will be evaluated on the parasite.

## 1. Introduction

Toxoplasmosis is a zoonosis parasitic disease with worldwide prevalence which caused by an obligate intracellular protrozoan; *Toxoplasma gondii*. Clinical congenital manifestation of toxoplasmosis is abortion and premature birth, hydrocephalus, microcephaly, jaundice and also chorioretinitis, anemia, pneumonia, and intracranial calcification in infants. Toxoplasmosis can also cause severe disease in individuals with immunodeficiency such as HIV-positive and cancer and transplant patients due to treatment with immunosuppressive drugs [1–4].

The current treatment in toxoplasmosis associated a range of side effects such as toxicity in pregnancy and bone marrow suppression, requirement of long courses, and lack of effect on the parasite cystic forms [5]. Due to the toxicity of chemical drugs and people's tendency to use traditional drugs and routs, many studies have been done on medicinal herbs and traditional methods in the world [6]. *Acorus calamus* (sweet flag) has used as traditional medicinal herb in China and India. The American native people, the dried root and its powder, put into up the nose for inhibition of inflammation of the mucous membrane [5, 7]. The other pharmacological properties of *A. calamus* contain anti-inflammatory, antipyretic, antidiarrhoeal, antimutagenic, anticellular and immunosuppressive, larvicidal, antimicrobial, antiulcer, and cytoprotective [8]. The rhizomes and leaf oils of *A. calamus* have been reported from the lower Himalayan region of India, with the major compounds in the rhizome oil, while *β*-asarone and linalool in the leaf oil [9]. Joshi et al. showed the essential oil of *A. calamus* and its major compound *β*-asarone that has a bactericidal property against pathogen bacteria and fungi [10]. Anthelmintic and antibacterial properties of rhizome are probably belonged to phenylpropanoid *β*-asarone [11]. This study was conducted to determine effects of ethyl acetate extract of subterranean parts of *Acorus calamus* on *Toxoplasma gondii* in vivo and in vitro.

## 2. Material and Methods

### 2.1. Ethics Approval

The present study is based on guidelines for the care and use of laboratory animals [12]. The Ethics Committee of Animal Experiments of the Shiraz University of Medical Sciences approved this research project (permit number IR.SUMS.REC.1398.355).

### 2.2. Collection of Samples

Several batches of rhizome of the fresh plant of *Acorus calamus* were prepared from regions of high altitude of forest in Sari city, northern Iran, in March 2015. Identification was carried out in Faculty of Pharmacy, Shiraz University of Medical Sciences. The parts of the plant were dried and grinded to powder. The plant herbarium specimens are collected in Payam Noor University of Mazadaran, and the dried rhizomes samples (Number: MPRCM-94-87) are maintained in Medicinal Plants Processing Research Center, Shiraz University of Medical Sciences.

### 2.3. Extraction

Rhizomes of the plant were dried in shade (26°C, 2 weeks). To prepare the ethyl acetate extract, 30 g powder of the dried parts was poured in dark sterilized kartush containing 600 ml of ethyl acetate. The extract was provided using Soxhlet extractor. Each step of Soxhletation elongated approximately 6 hours. The extraction was concentrated with rotary evaporator apparatus and then dried by speed vacuum during 48 hours. The extract was maintained in dark bottle at 2-8°C condition.

### 2.4. Parasites

The virulent RH strain of *T. gondii* was obtained from Tehran University of Medical Sciences, Tehran, Iran. Tachyzoites of the RH strain of *T. gondii* were maintained by serial intraperitoneal passaging in BALB/c inbred mice. After 72 hours, 10^6^ parasite inoculation in the mice, the tachyzoites were collected after repeated flushing of the peritoneal cavity by phosphate buffered saline (PBS) at a pH of 7.2. Then, tachyzoites were harvested and centrifuged for 5 min at 200 g at room temperature to remove peritoneal cells and cellular debris. The supernatant was collected and centrifuged for 10 min at 800 g [1]. The pellet, enriched with parasite tachyzoites, was recovered with PBS and used in the experiments.

### 2.5. Extracellular Viability Assay

We dissolved the extract in DMSO and then PBS to obtain a final concentration of 10 mg/ml. The final concentration of DMSO should not exceed 1%. Various concentrations (25, 50, 100, 200, 400, 800 *μ*g/ml) of the extract were then prepared by the following: 2.5-80 *μ*l of the final concentration was added to 920-997.5 *μ*l of PBS that contained 2 × 10^5^ tachyzoites. Tachyzoites were incubated with either DMSO (as control) or the diluted compounds for 1.5 h at 4°C. Next, the tachyzoites were collected in Eppendorf tubes and incubated for 30 min at 4°C with 50 *μ*g/ml propidium iodide (PI, Sigma Company, USA). After incubation, the parasites were kept on ice and in the dark until analysis. Positive controls for PI staining were acquired by incubating parasites in the presence of 0.2% saponin. The cell suspension was transferred into polystyrene flowcytometry tubes (BD Falcon Company, USA). We performed data acquisition and analysis, with a FACS Calibur flow cytometer (Becton-Dickinson, San Jose, USA) and Cell Quest Pro software. A total of 1000-30000 events were acquired in the region that had been previously established as corresponding to the parasites [13]. All of the tests were undertaken in duplicate.

### 2.6. In Vivo Experiments

In this study, a total of 9 groups including 10 BALB/c inbred mice were considered. 2 × 10^5^ tachyzoites were intradermally inoculated into 8 groups including 10 mice. Based on results of in vitro experiments, 6 groups, doses 32, 64, 128, and 256 mg/kg, were orally, and doses 128 and 256 mg/kg, intraperitoneally administered: VII—received sulphadiazine as positive control and VIII—received PBS including DMSO 1%. These concentrations were daily administrated 24 hours after inoculation due 10 days continuously. Mice were followed for 15 days after inoculation. If the mice died, their liver touch smears were stained with Giemsa stain and observed under light microscopy for parasite detection [13].

### 2.7. The Acute Toxicity Assay of the Extract

For toxicity evaluation of this extract, a group only received a maximum dose of (256 mg/kg). Then, the mice were followed for any manifestation including paw licking, stretching of the entire body, salivation, weakness, respiratory distress, sleep, coma, and death in the first four hours and subsequently daily for 14 days.

### 2.8. Assessment of the Total Phenolic Content in the Extract

The total phenolic content of the extract was measured by the Folin–Ciocalteu method and Folin's phenol reagent. In this experiment, garlic acid was used as standard. First, a serial dilution of garlic acid (0.024, 0.03, 0.075, 0.105 mg/ml) was provided by methanol and then filtered by Whatman Grade 1 filter paper. 0.5 ml of the different concentrations was diluted by 2.5 ml of Folin–Ciocalteu reagent and 2 ml of 7.5% (w/v) sodium carbonate in 20°C. Absorbance was measured at 765 nm. All of the tests were undertaken in triplicate, and calibration curve was drawn. 500 *μ*L of the crude extract was diluted by 2.5 ml of Folin–Ciocalteu reagent and 2 ml of 7.5% (w/v) sodium carbonate in 20°C. Absorbance was measured at 765 nm. The total phenolic content was calculated from the calibration curve.

### 2.9. Total Flavonoid Content

250 mg of dried powder was diluted with 20 ml and sonicated for 15 minutes. The extract was filtered, and 5 ml of it was mixed with 5 ml of 2% (w/v) AlCl_3_ solution for 15 minutes in dark condition. Absorbance was measured at 415 nm. The total flavonoid content was calculated from a calibration curve obtained from quercetin.

### 2.10. Data Analysis

Data were gathered in SPSS software (version 16, Chicago, USA). In vitro results were analyzed by the Kuruskal–Wallis and Spearman correlation tests, whereas Kaplan-Meier and log rank (Mentel-Cox) were used in vivo. *P* < 0.05 was considered statistically significant.

## 3. Results

In this study, the mortality of *Toxoplasma* tachyzoite cells exposed to the different concentrations of ethyl acetate extract of *Acorus calamus* was measured using the flowcytometry technique.

**Figure 1:**
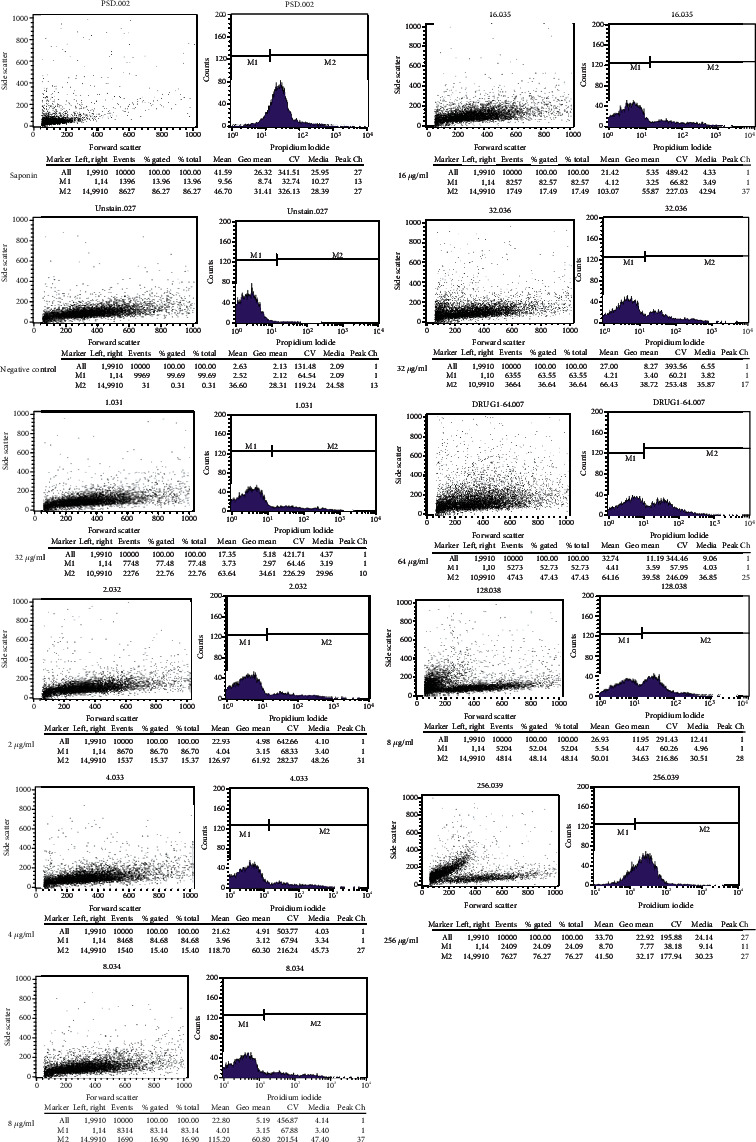
Flowcytometry results of the mortality rate of tachyzoites exposed to different concentrations of *Acorus Calamus* extract using propidium iodide.

**Figure 2:**
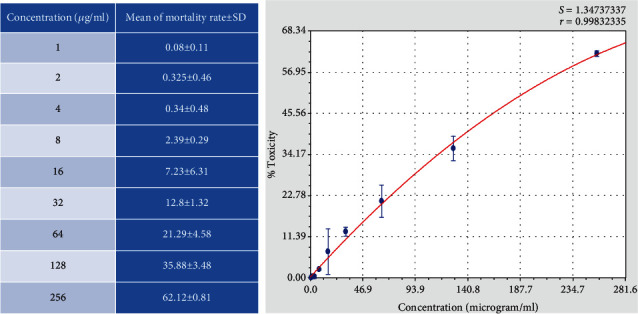
Flowcytometry results of the mortality rate of tachyzoites exposed to different concentrations of ethyl acetate extract of *A. calamus.*

Figures 1 and 2 show that there is an inverse relationship between the concentrations and mortality rate of the parasite tachyzoite. More than 62 percent of *Toxoplasma* tachyzites were killed at maximum concentration (256 *μ*g/ml). IC_50_ of the extract on the parasite was calculated 200.01 ± 7.74 (*μ*g/ml).

In vivo results showed a significant difference at maximum concentration (256 mg/kg) with gavage administration (*P*˂0.031), whereas peritoneal inoculation of this concentration did not effect on longevity of the mice.

Any signs of physical changes that belonged to the toxicity of the extract in the tested animals were not detected, and the entire mice group was live (Table 1).

**Figure 3:**
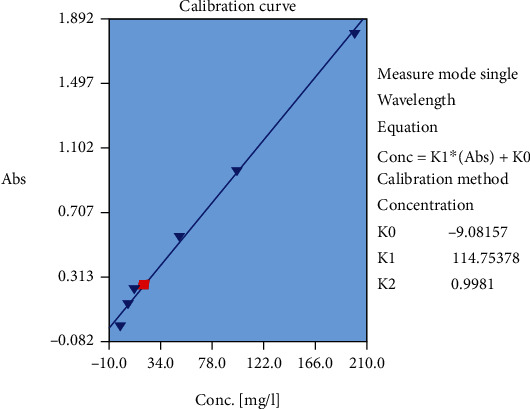
Assessment of the phenolic compound content of ethyl acetate extract of *A. calamus* based on standard graph of gallic acid.

Figure 3 shows the phenolic compound content of the extract based on standard graph of gallic acid. The plant extract was rich of phenolic compounds (41.27 ± 0.21 mg/g).

**Figure 4:**
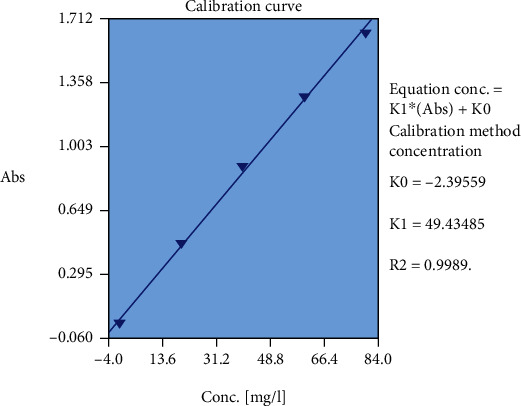
Assessment of the flavonoid compound content of ethyl acetate extract of *A. calamus* based on standard graph of quercetin.

Figure 4 shows the flavonoid compound content of the extract based on standard graph of quercetin. The flavonoid compound content of plant extract was 4.79 ± 0.01 mg/g.

## 4. Discussion

*Acorus calamus* is consumed in traditional medicine, and its rhizomes are widely used to subside in clinical signs such as chronic diarrhea, dysentery, fever, and rheumatism [8]. Also, it is used traditionally in the treatment of various ailments including neuralgia, dyspepsia, kidney and liver troubles, eczema, sinusitis, asthma, bronchitis, hair loss, and other disorders [14].

In vivo results of our experiments on the extract toxicity showed that any clinical signs were not occurred in the tested animals. Similarly, Muthuraman et al. showed that the high doses of the hydroalcoholic extract of the plant rhizome could not create any toxic effects in rats [15]. Another study has shown that the high dose of the extract is very well tolerated in rodents but it is associated with a mild elevation in levels of the liver enzymes [16].

In our study, the ethyl acetate extract of the rhizome was rich of phenolic compounds. It has commonly been assumed that the plant pharmacological fundamentals such as antidepressant, antianxiety, anti-Alzheimer's, anti-Parkinson's, antiepileptic, anticancer, antihyperlipidemic, antithrombotic, anticholestatic, and radioprotective activities were related to phenolic compounds such as *α* and *β*-asarone molecules. Alpha and beta asarones are chemical compounds of the phenyl propanoid class [14, 17, 18]. Isoeugenol, another phenolic compound, is found in *A. calamus* leave samples [19].

Oliveira et al. showed that the mortality in mice with toxoplasmosis can be inhibited by phenolic compounds such as vanillin [20]. Moreover, Choi et al. indicated that the phenolic compounds of ginger root extract can inactivate apoptotic proteins in host cells infected to *Toxoplasma.* Remarkably, the proteins inhibit secretion of inflammatory cytokines in vivo [21].

In this study, we measured a low content of the flavonoid compounds in the plant extract, but in other studies, apigenin, luteolin, and diosmetin as flavonoid compounds were found in *A. calamus* [22]. Mac Laren et al. showed that some flavonoids, such as apigenin and genistein, can inhibit *Toxoplasma* growth due to inhibition of protein tyrosine kinase [23]. Similarly, other flavonoid compounds such as naringenin and genistein had the notable activities against *Cryptosporidium* in cell culture [24].

Quercetin as a flavonoid compound can inhibit synthesis protection factors such as Hsp90, Hsp70, and Hsp27, and consequently, *Toxoplasma* remains sensitive due to the effects of host immune responses [25].

Lehane and Saliba showed that certain common dietary flavonoids especially luteolin can inhibit the intraerythrocytic growth of the chloroquine-sensitive and chloroquine-resistant strains of *P.* falciparum [26].

In our study, the direct effect of the different concentrations of ethyl acetate extract of *A. calamus* on *Toxoplasma* tachyzoite was measured using the flowcytometry technique. In this technique, the propidium iodide, a fluorescent DNA-binding dye, is used for the evaluation of dying cells. The results showed an inverse relationship between the concentrations and the mortality rate of the parasites.

In vivo results showed a significant difference at maximum concentration with gavage administration, whereas peritoneal inoculation of this concentration did not effect on longevity of the mice. However, the effect of peritoneal administration of concentration 128 *μ*g/ml was better than oral concentration.

## 5. Conclusion

Our study demonstrated that *Acorus calamus* extract had significant activities against *T.gondii* in vivo and in vitro which may be connected to high amount of phenolic compounds. We suggest that the effects of the various fractions of this extract on the parasite are investigated. Alternatively, the administration types and dosage of the extract on the parasite must be evaluated.

## Figures and Tables

**Table 1:** In vivo results of different administrations of ethyl acetate extract of *Acorus calamus* in treatment of murine toxoplasmosis.

Administration		Time of death (postinoculation day)						Mean of longevity
		6	7	8	9	10	11	
Ethyl acetate extract of *Acorus calamus*	32 mg/kg (oral)		^∗∗∗∗∗^	^∗∗^	^∗∗^	^∗^		7.9
	64 mg/kg (oral)	^∗∗^	^∗∗∗∗∗∗^		^∗^	^∗^		7.3
	128 mg/kg (oral)	^∗∗^	^∗∗∗∗∗^	^∗∗^	^∗^			7.2
	128 mg/kg (intraperitoneal)		^∗^	^∗∗∗∗∗^	^∗∗∗^	^∗^		8.4
	256 mg/kg (oral)			^∗∗∗^	^∗∗∗∗∗∗^		^∗^	8.9
	256 mg/kg (intraperitoneal)	^∗∗∗∗^	^∗∗∗^	^∗∗^	^∗^			7
	256 mg/kg (oral) without parasite	**—**	**—**	**—**	**—**	**—**	**—**	—
Sulfadiazine (30 mg/kg) as positive control		**—**	**—**	**—**	**—**	**—**	**—**	—
Negative control			^∗∗∗∗^	^∗∗∗^	^∗∗∗^			7.9

^∗^ shows death event.

## Data Availability

The data used to support the findings of this study are available from the corresponding author upon request.
